# A commentary on the practice of integrated medical curriculum in the interdisciplinary field of medical engineering

**DOI:** 10.1080/07853890.2022.2050421

**Published:** 2022-03-11

**Authors:** Peng Zhang, Liang Ji, Guomin Zhou, Xuan Yao

**Affiliations:** a School of Medicine, Shanghai University, Shanghai, China; b School of Medicine, Shanghai Jiao Tong University, Shanghai, China; c Shanghai Medical College, Fudan University, Shanghai, China

**Keywords:** Medical education, Interdisciplinary, integrated curriculum, medical engineering, graduate student

## Abstract

Shanghai University School of Medicine was a newly established medical college in 2018. It is founded on the national health development policies, international medical development trends and the close relationship between the advantages of new medical courses and medical artificial intelligence, it is dedicated to using intelligent medicine as the breakthrough point, training graduate students in two interdisciplinary medical engineering subjects as the priority, and implementing the integrated medical curriculum teaching reform. In this paper, we introduce the background of the integrated medical curriculum system at the Shanghai University School of Medicine, the horizontal and vertical integration of medical courses in interdisciplinary medical engineering subjects, the cross-integration of traditional integrated medical courses with other disciplines and specialties, and the transformation mode of medical science and technology innovation led by artificial intelligence under the support of three-dimensional curriculum integration, putting forward the prospect of the curriculum integration system and providing experiences and references for other medical schools.

**KEY MESSAGES**
- This paper introduces the necessity and feasibility of implementing integrated medical course teaching in a newly established medical college.
- This paper introduces the strategies and concrete measures we took to implement integrated medical course teaching.
- The analysis of examination papers and other evaluations revealed that the integrated medical teaching for graduate students with non-medical professional backgrounds is feasible.

## Introduction

In September 2008, the Ministry of Education and the Ministry of Health of China issued the Medical Undergraduate Education Standards—Clinical Medicine Specialty which explicitly required medical colleges and universities to carry out vertical and/or horizontal curriculum reform and reasonably integrate curriculum contents [1–3]. Since then, the integrated curriculum has received increasing attention from colleges and universities. To meet the needs of medical innovation and development, Shanghai University established the School of Medicine (SUSM) at end of 2018 and took the lead in setting up two graduate training programs closely related to biomedicine, including *Intelligent Medical Diagnosis and Treatment,* and *New Drugs and New Materials*. Implementing a practical and effective integrated medical curriculum system is critical to achieving the goal of training graduate students and provides a theoretical and practical basis for the follow-up reform of the integrated medical curriculum at SUSM.

In 1952, the Case Western Reserve University School of Medicine established an organ-system-oriented comprehensive project teaching model. The integrated medical curriculum has been studied in China since the 1990s. Considering the subject attributes and teaching time of the integrated curriculum, there are two modes of organ and system integration courses: horizontal and vertical integration. Horizontal integration refers to the integration of parallel subjects in the learning stage, the most common being the integration of basic biomedical subjects and clinical subjects. Vertical integration refers to the integration of fudamental biomedical disciplines and clinical disciplines across traditional learning stages [4]. It constantly deepens the understanding and application of essential knowledge through clinical scenarios, breaking the teaching framework of traditional basic and clinical medicine. When the two integration models are combined, a spiral integration is obtained, which spans the disciplinary attribute and traditional learning stage. The teaching of fundamental biomedical science and clinical science runs through the entire learning process [4]. After nearly 70 years of development in China, the integrated medical curriculum teaching model has been recognised as an effective way to reconcile the explosion of medical knowledge with limited learning time, helping students develop a holistic view of medicine and improve their clinical thinking ability to solve problems comprehensively. Since the establishment of SUSM in 2018, we have initiated curriculum reform of the graduate training program, which integrates relevant disciplines such *biology*, *basic medicine*, and *clinical medicine*, and constructed a curriculum system with both horizontal and vertical integrations.

The integrated teaching reform was formally implemented in April 2019 under the auspices of the dean in charge of teaching at SUSM. The reform was conducted through joint research and discussion with all faculty at SUSM and from relevant departments within and outside Shanghai University, supported by the Shanghai University-funded project of “Construction of First-class Graduate Education Industry Education Integration Talent Training Base.” This paper expounds on the design background, scheme, and implementation of the Integrated Medical Curriculum system in SUSM. Furthermore, it advances the prospects of the system, hoping to provide a reference for the training and teaching reform in other medical schools.

## Background of the integrated medical curriculum in Shanghai University School of Medicine

Shanghai University established a first-class research-oriented medical college based on the advantages of science and engineering subjects in response to development needs. However, in the context of several well-known medical colleges in Shanghai, accelerating their development of medical colleges is a real problem faced by Shanghai University. According to the development of health policy [1–3], Shanghai University decided to use intelligent medicine as the breakthrough point, prioritising training graduate students in two interdisciplinary medical engineering subjects and cultivating high-level medical talents as the goal, to gradually integrate their medical college into the development path of mainstream medical disciplines.

As mentioned above, the integrated medical curriculum is a set of learning systems for medical students that conform to the development direction of integrated medicine. It has drawn increasing attention and has been widely promoted [4,5]. However, it is primarily implemented in undergraduate medical education and rarely mentioned in graduate medical and industrial cross-specialty education. As it is known, graduate education aims to develop high-end teaching and research abilities. With limited medical education background and schooling time (generally 3 years), the implementation of the integrated medical curriculum at SUSM enables graduate students to acquire the necessary basic and clinical medicine knowledge, and creates favourable conditions for conducting intelligent medical research. The combination of biomedicine and artificial intelligence requires a horizontal integrated medical curriculum, whereas the complexity and reserve demand of medical knowledge require a vertical integrated medical curriculum. Therefore, we conducted the teaching reform of the integrated medical curriculum in medical and industrial cross-specialties for graduate students at SUSM.

## Integrated medical curriculum in the Shanghai University School of Medicine

We learned from the American pre-medical education model, which prioritises science and engineering foundations over medical practice [6]. We leveraged the strengths of Shanghai University to explore a new talent training model and new integrated medical courses. First, to facilitate the implementation of integrated medical curriculum, we established two new interdisciplinary platforms, namely, the Medical-Science-Engineering Cross-Platform and the Medical-Teaching-Research Cross-Platform. The former platform primarily comprises faculty members with medical knowledge and mathematical and engineering foundations. The latter platform primarily comprises faculty and staff members who can apply cutting-edge intelligent technologies to medical innovation and transformation, ranging from the medical and industrial intersection to medical and industrial integration. Second, to maximise the use of teaching and research resources within and outside the SUSM to construct an in-depth integrated medical curriculum, we built a specialised talent training platform in collaboration with relevant colleges, hospitals, and research institutes within and outside the university. For example, we successively signed a comprehensive cooperation agreement with the School of Computer Automation of Shanghai University and jointly constructed a Medical Imaging Teaching and Research Base of Shanghai University with the Shanghai Universal Medical Imaging Diagnostic Centre, a teaching hospital with the Shanghai Public Health Centre, a clinical medical school with the Shanghai Pudong Public Welfare Hospital and a teaching hospital with the Shanghai Dermatological Hospital.

**Figure 1.**
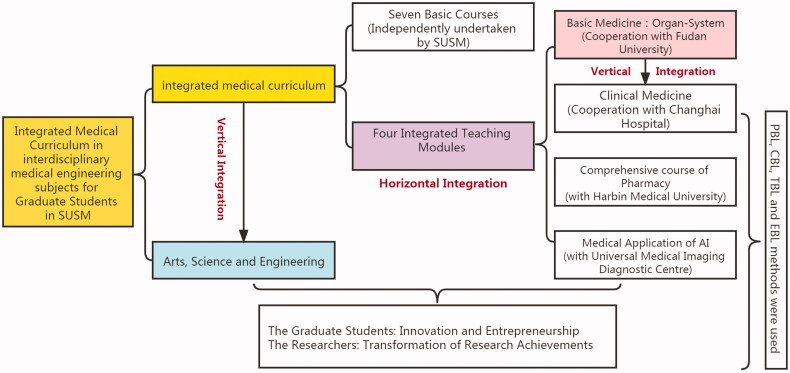
Schematic diagram of integrated medical curriculum in SUSM.

At present, four integrated teaching modules have been established for the graduate training programs of *Intelligent Medical Diagnosis and Treatment* and *New Drugs and New Materials*: *Basic Medicine*, *Clinical Medicine*, *Comprehensive Course of Pharmacy and Artificial Intelligence Medical Application* (Figure 1).

The integrated *Basic Medicine* course was co-established with the Shanghai Medical College of Fudan University and is divided into two modules. First, courses related to *basic medicine* were horizontally integrated into seven teaching units that include essential medical knowledge, such as *molecular biology* and *cell biology*, *human development*, *basic structure and physiological function of organ systems*, *pathology* and *basic methods* and *principles of treatment*.Subsequently, three teaching units were set up to vertically integrate clinical medicine, diseases and treatment of major systems. The three integrated teaching modules of *Clinical Medicine*, *Comprehensive Course of Pharmacy* and *Artificial Intelligence Medical Application* were designed and implemented in cooperation with Changhai Hospital affiliated with Naval Military Medical University, Harbin Medical University, and Shanghai Panoramic Medical Imaging Diagnosis Centre, respectively. Shanghai University uses a third-party evaluation (the Mycos Report) to assess the quality of teaching. To a certain extent, the combination of horizontal and vertical integration reduces students’ learning pressure, save teaching resources, and improves learning and teaching efficiency [7,8].

SUSM focuses on intelligent medicine and has jointly established the Medical Imaging Teaching and Scientific Research Base of Shanghai University with Shanghai Universal Medical Imaging Diagnostic Centre, the practice and research base for graduate students majoring in *intelligent medicine diagnosis and treatment*. The Shanghai Universal Medical Imaging Diagnostic Centre has enormous clinical cases and practical experience. During or after the course study, graduate students can access the frontline of medical imaging to understand clinical needs and find problems, bring these problems back to school for research, and then transform the research results and apply them to the practice of medical imaging. It will help them acquire knowledge quickly and comprehensively [9–11]. The graduate program is a journey of exploration, discovery, and creation between the tutor and the student. To improve the quality of interdisciplinary graduate training and enhance scientific research and innovation ability, SUSM established the Interdisciplinary New Medical Graduate Innovation Fund Project in 2019. The application, evaluation and management of the project should be conducted through fair competition and assessment. The selected project should be determined after the application of the graduate student, recommendation of the supervisor and expert evaluation.

### Preliminary effect of the implementation of medical integration curriculum

Since April 2019, SUSM has been exploring the implementation of integrated medical curriculum for graduate students majoring in interdisciplinary medical engineering and has achieved preliminary results. The following is an analyses of the teaching effects, taking the students’ academic achievements in 2020 as an example.

There were 43 master’s and 15 doctoral students who participated in the study, including 38 students majoring in *new drugs and materials* and 20 students majoring in *intelligent medical diagnosis and treatment*. We used the same examination paper for testing because they were studied together.

**Figure 2.**
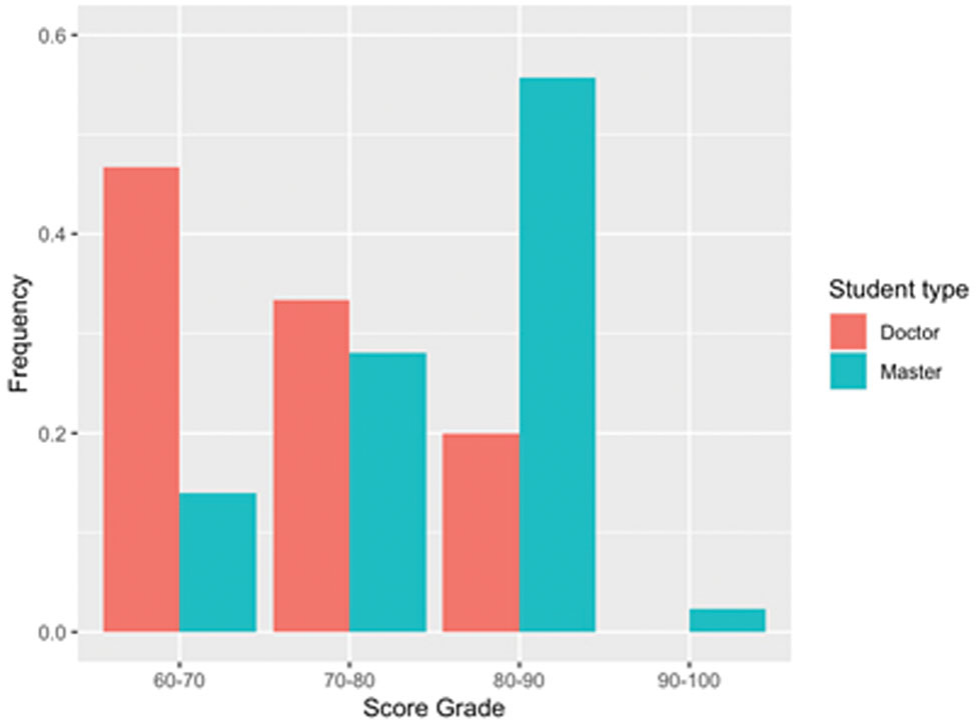
Frequency distribution of scores for master‘s and doctoral students.

We analysed the final examination papers and created a frequency distribution diagram of the score segmentation and basic descriptive statistics (Figure 2, Table 1). The total score of master’s students was primarily distributed between 80 and 90, while the total score of doctoral students was primarily distributed between 60 and 80. From the score range of 60–90, we found that the frequency distribution of the master’s students demonstrated a increasing trend, while that of the doctoral students demonstrated a declining trend.

**Table 1.** Basic descriptive analysis index of final examination paper score.

	Minimum score	Maximum score	Mean	Standard deviation	Skewness	Kurtosis
Doctoral Students	60	82	71.47	7.259	−0.036	−1.215
Master’s Students	60	90	78.26	6.698	−0.701	0.210

Table 1 demonstrates that the minimum score for master’s and doctoral students was 60 while the maximum were 90 and 82, respectively. The mean of the master’s students (78.26) was higher than that of doctoral students (71.47). The standard deviation indicated that the dispersion degree of the two types of students in this test was moderate, and the dispersion degree of students’ scores was acceptable, indicating that the questions and students’ level of the test paper were normal. Meanwhile, the skewness values of the scores of master’s and doctoral students were −0.701 and −0.036 respectively, indicating that the total score distribution of the two types of students was skewed to left. The kurtosis values of the total score of master’s and doctoral students were 0.210 and −1.215, indicating that the total score distribution of the master’s students is steeper than that of the doctoral students. The overall descriptive statistics of the distribution of the two types of student total scores indicated that they were normally distributed and within an acceptable range.

**Figure 3.**
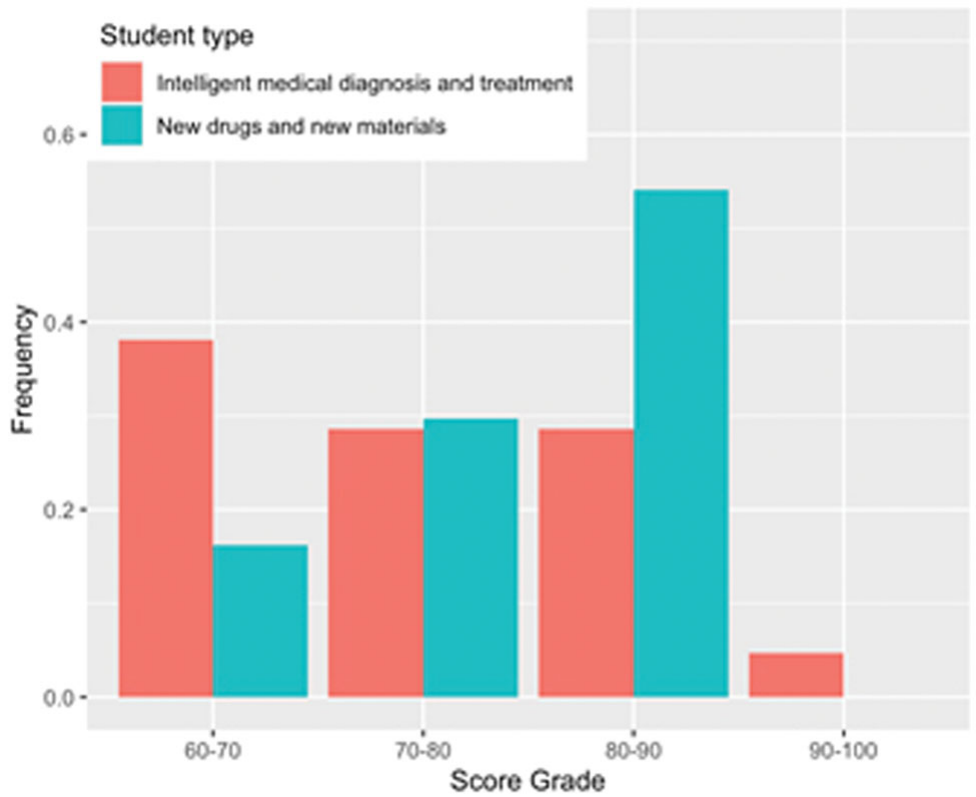
Frequency distribution of scores for all students in two majors.

Figure 3 is illustrates the frequency distribution of scores of students in the majors. As can be seen, the score of students majoring in *new drugs and new materials* increases gradually, while the score of students majoring in *intelligent medical diagnosis and treatment* is relatively consistent. This indicates that students majoring in *intelligent medical diagnosis and treatment* are relatively average overall, while those majoring in *new drugs and new materials* account for most students with high scores.

Based on the above analyses, we believe that graduate students majoring in interdisciplinary medical engineering initially met our expected goal through learning an integrated basic medical curriculum, which means that they mastered the fundamental knowledge of *basic medicine* in a relatively short time. For graduate students without a background in medical education, it is very challenging to master basic and clinical medical knowledge during a brief period of schooling (usually three years). Doctoral students need to spend more energy on research than master’s students, who are primarily concerned with course work. Therefore, doctoral students have limited time to study after class, resulting in a slightly lower score than master’s students. The above results indicate that the implementation of an integrated teaching model in the graduate teaching of medical engineering cross-discipline is feasible and effective; however, the model needs to be further improved in terms of reasonably adjusting class hours, mobilising students' learning enthusiasm, and improving teaching quality.

### Integration of medical curriculum and teaching ideas and methods of medical and engineering inter-specialty

The dynamic development trend of *Precision Medicine* model is emphasized. *Precision Medicine* has always been based on gene testing, massive medical data processing and targeted drug therapy [12,13]. The model of *Precision Medicine* has changed with the development of digital technology and other new technologies, such as wearable devices, the Internet, and 5 G mobile communications. For example, the medical scene has expanded beyond hospitals to include patient families, resulting in the development of personalised precision medicine [14]. The organic fusion of big data technology and medical image diagnosis produced new imageomics methods. Controlling surgical robots will transform the traditional medical instruments used by doctors into the human-machine cooperation mode. At present, the application and development of artificial intelligence in medical imageomics is a product of the synergistic development of the medical industry. The program of intelligent medical imaging at SUSM focuses on precision imaging, diagnosis of diseases and specialised medical treatment.

In the future, Shanghai University will strengthen the resource integration in *Precision Medicine* and other research fields through integrated medicine courses, increase the investment in cooperation with relevant nuclear medicine molecular imaging equipment manufacturers or enterprises, and jointly build molecular imaging centre. Precision medical treatment and medical integration teaching are incorporated into a knowledge system. Through integration, a new knowledge system resembling LEGO blocks can adapt to the increasing medical knowledge. Mutual matching modules can be configured arbitrarily, where new modules can be added at any time. The system does not operate in the same way as traditional disciplines education, which is based on a jigsaw puzzle where modules cannot be added or removed or their positions adjusted [15].

## Application of PBL, CBL and EBL in integrated medical curriculum

As mentioned previously, SUSM has integrated new medical teaching ideas and methods based on extensive research [16–20]. Problem-based learning (PBL) and case-based learning (CBL) teaching methods will always be applied in the integration module of clinical and intelligent medicine. PBL teaching in integrated medical courses can be used effectively at various teaching stages of basic, basic-clinical, and clinical teaching. This teaching method complements integrated medical courses well, and its numerous advantages contribute to improving students’ self-study, analytical and comprehensive ability, written and oral expression ability, and teamwork ability [21].

SUSM has used artificial intelligence technology to empower medicine, developed a new medical concept for integrated medicine curriculum, and emphasised the crucial role of translational medicine [22,23]. Supported by the interdisciplinary disciplines of medicine and industry, SUSM jointly cultivates high-end talents with Shanghai Medical Imaging Centre and promotes the integration of medical education, research, development and application, and development of translational medicine through the organic combination of high-level basic discipline research and clinical application research, technology transformation and industrial production. Engineering technologies, including electronic technology, computer technology, Internet and Internet of Things (IoT) technologies, machine learning, pattern recognition and artificial intelligence, brain machine interface and human-computer interaction technology, medical imaging, biomedical signals, medical examination, medical information, medical diagnosis, are always critical for the cross-docking demand on the road of scientific research. Improving the effectiveness of diagnosis and treatment and reducing the cost of treatment are the ultimate goals of medicine and engineering.

At present, there are teachers and graduate students in the cooperation project, which comprise medical and industrial doctoral supervisors and joint training supervisors, engineering teachers, and medical teachers who collaborate to lead graduate students to tackle problems together and develop solutions. Graduate students receive training in the project from the start of enrolment.

Evidence-based learning (EBL), as a knowledge system for integrated medical courses, is particularly crucial in the modules of integrated medical and artificial intelligence courses. As graduate students come from various universities and their bachelor’s degrees do not involve evidence-based medicine [24,25], it is a real educational challenge to introduce evidence-based medicine at an early stage in medical school and emphasise its importance in a physician's career. SUSM proposes the goal of medical applications for AI and integrated courses. It collaborated with the Shanghai Panorama Medical Imaging Diagnostic Centre to develop an intelligent medical data platform, which serves as a multimodal, all-round support for real-world research and can provide powerful data visualisation, data exploration, and machine learning tools, discover clinical problems, improve the efficiency of clinical research, and maintain the level of scientific development. Collaboration can promote the improvement of clinical teaching and training levels and accelerate subject development, and reliable scientific information facilitates scientific decision making.

## Integrated medicine curriculum development prospect at Shanghai University School of Medicine

We compiled the following points to develop an integrated medicine curriculum system in medical colleges and universities.

1. Avoid treating medical problems in isolation and emphasise the integration of traditional Chinese and Western medicine. Treatment and prevention should not be separated [25].
2. Strengthen theoretical research on integrated medicine. The SUSM will strengthen the integration of medical practices in the future. It could take the following form: holding an all-around strategic cooperation ceremony and a high-profile medical-industrial forum. Cooperation between universities, universities, and enterprises can be employed in an embedded way to achieve all-around in-depth cooperation, including establishing an integrated medicine academic organization and teaching guidance committee, compiling professional journals of integrated medicine, publishing integrated medicine series, textbooks, or monographs, and setting up integrated medicine institutes [26,27].
3. In an integrated curriculum-based interdisciplinary program, students will access joint degree programs with other schools, such as MD-PHD, MD-MPH, MD-MBA and MD-MPP, to ensure efficient and accurate integration of knowledge.

## Conclusion

The application of traditional integrated medical courses to traditional medical teaching has matured. Medical colleges and universities can fully integrate medical courses according to their teaching requirements by matching their corresponding teaching methods. SUSM will take new medical subjects as the lead to cultivate excellent medical scientists, focussing on the implementation of “5 + 2” working steps, namely, “set up a forum, set up a fund, form a mechanism, establish a set of system, built a strong team, and construct featured courses to establish a number of practice bases,” to further promote the training of innovative talents in new medical sciences and the construction of new integrated medical disciplines.

As most graduate students come from various non-medical majors, it is difficult for them to master basic and clinical medical knowledge in a limited time if traditional teaching mode is adopted. In such a complex situation, the integration of medicine, medical engineering, and intelligence courses, and the extensive use of PBL, CBL, EBL, and other teaching methods are used to build a new integrated medical curriculum system resembling LEGOs, which is an extremely intelligent integrated medical curriculum that has been adapted to modern intelligent medicine. This requires the scientific integration of subjects, follow-up of teaching methods and examination forms, and assurance of logistics work. Convergence of disciplines transforms scientific research into a new paradigm and provides new opportunities for breakthroughs in key technologies. The new concept of integrated medical course collects and integrates a large amount of multi-dimensional data from the real world and research subjects and relevant clinical, biometrics, individual behaviour, and follow-up, among other things, focussing on the ability to acquire knowledge from large and complex data. Medicine and industry each have their distinct advantages. With the advancement of research, only “cross-field” and extensive collaboration can propel the cross-discipline of medicine and industry foward.

## Data Availability

The datasets supporting the findings of this study are openly available at 4TU.ResearchData at 10.4121/16442301.
